# Genome-wide analysis of the G-box regulating factors protein family reveals its roles in response to *Sclerotinia sclerotiorum* infection in rapeseed (*Brassica napus* L.)

**DOI:** 10.3389/fpls.2022.986635

**Published:** 2022-08-12

**Authors:** Qinfu Sun, Ying Xi, Panpan Lu, Yingying Lu, Yue Wang, Youping Wang

**Affiliations:** Jiangsu Provincial Key Laboratory of Crop Genetics and Physiology, Yangzhou University, Yangzhou, China

**Keywords:** 14-3-3, GF14, GRF, general regulatory factor, *Brassica napus*, *Sclerotinia sclerotiorum*, rapeseed, G-box regulating factors

## Abstract

The G-box regulating factors (GRFs) are involved in a wide array of signal transduction pathway and play important roles in plant physiological and developmental processes and stress responses. The GRF proteins have previously been described in several plant species, but not in rapeseed (*Brassica napus* L.). In this study, we carried out genome-wide analysis of *GRFs* in *B*. *napus* based on the available genome sequence information, and analyzed their expression in different tissues under different hormone treatments and after inoculation with *Sclerotinia sclerotiorum*. We identified 46 putative *BnaGRF* genes in rapeseed, unevenly distributed on 18 chromosomes. Like the 14-3-3 proteins in other plant species, the 46 putative BnaGRFs could be classified into two major evolutionary branches: epsilon (ε) group and non-epsilon (non-ε) group. Evolutionary analysis indicated that the *BnaGRF* gene family expanded in both groups much before speciation. We discovered an expansion of the *14-3-3* gene family that likely occurred during a recent gene duplication event. Collinearity analysis revealed that most of the *BnaGRF* genes shared syntenic relationships. Global gene expression profiling of *BnaGRFs* by RNA-seq analysis showed 41.3% (19/46) response to *S*. *sclerotiorum* infection, and this response was probably mediated through jasmonic acid (JA) and salicylic acid (SA) signaling pathways. These results provide key insights into the role of 14-3-3s in the biotic stress response and enhance our understanding of their multiple functions in *B*. *napus*.

## Introduction

The 14-3-3 proteins were named in 1967 during an extensive study on bovine brain proteins, which were given numerical designations based on the results of column fractionation and electrophoretic mobility assays (Rosenquist et al., 2001). The 14-3-3 proteins are present in all eukaryotic organisms and are expressed in virtually all tissue types (DeLille et al., 2001). Initially, an *Arabidopsis thaliana* isoform was identified as part of a protein/G-box complex, and therefore named “G box factor 14-3-3” (GF14), and the 14-3-3 isoforms in plants were named G-box regulating factors (GRFs) (Rosenquist et al., 2001). The 14-3-3 proteins participate in several peculiar functions, such as the regulation of primary metabolism, ion transport, cellular trafficking, chloroplast, and mitochondrial enzyme activities (Camoni et al., 2018). Based on gene structure, the encoded plant 14-3-3 proteins are divided into two distinct groups, namely, epsilon (ε) and non-epsilon (non-ε) (Ferl et al., 2002).

An increasing body of evidence suggests that 14-3-3 proteins play an important role in different aspects of plant hormone physiology (Camoni et al., 2018). Research results show that 14-3-3 proteins interact with brassinosteroid (BR) receptor Brassinosteroid-Insensitive1 (BRI1), BRI1 Kinase Inhibitor (BKI1), BRI1 Suppressor phosphatase (BSU1), and the transcription factors BRI1-EMS-Suppressor1 (BES1), and Brassinazole-Resistant1 (BZR1), which act both as negative and positive regulators of BR signaling pathways (Chang et al., 2009; Ryu et al., 2010; Wang et al., 2011). The 14-3-3 proteins function as members of the transcriptional complexes of abscisic acid (ABA)-regulated genes. In embryonic barley roots, 14-3-3 proteins function in the ABA-regulated transcriptional cascade by interacting with bZIP transcription factors ABI1-3 and ABI5 (Schoonheim et al., 2007, 2009). Additionally, 14-3-3 proteins have been reported to regulate the cell expansion by stimulating the H^+^-ATPase-dependent proton extrusion into the cell wall, which was IAA-dependent regulatory processes. In Arabidopsis, IAA promotes Thr^947^ phosphorylation and subsequent 14-3-3 binding to H^+^-ATPase, and is responsible for its activation (Takahashi et al., 2012). 14-3-3 proteins interacted with components of the signaling pathways of gibberellic acid (GA) (Ito et al., 2014), jasmonic acid (JA) (Holtman et al., 2000), salicylic acid (SA) (Yang et al., 2009), and other plant hormones (Jaspert et al., 2011; Yoon and Kieber, 2013; Camoni et al., 2018) as well.

The 14-3-3 proteins participate in many physiological processes of plants, including flowering and chloroplast, stomatal, and root development. In rice, 14-3-3 proteins interact with the FT homolog Hd3a and the OsFD1 transcription factor in the apical cells of shoots to induce the transcription of *OsMADS15*, leading to flowering (Taoka et al., 2011; Kaneko-Suzuki et al., 2018), while in Arabidopsis, the 14-3-3 ν and μ proteins affect photoperiodic flowering by interacting with CONSTANS, a central regulator of the photoperiod pathway (Mayfield et al., 2007), and regulate root growth and chloroplast development as components of the photosensory system (Mayfield et al., 2012).

Whole-genome studies and reverse genetics approaches revealed that 14-3-3 proteins are involved in plant stress resistance. Genome-wide analysis of 14-3-3 proteins in rice (Chen et al., 2006), soybean (Wang et al., 2019), wheat (Shao et al., 2021), grape (Cheng et al., 2018), and other crops showed that the expression of some 14-3-3 family members is induced, while that of others is repressed by stresses. Overexpression of a wild soybean (*Glycine soja*) 14-3-3 gene, *GsGF14*o**, in Arabidopsis caused deficits in root hair formation and development, thereby reducing the water intake capacity of the root system (Sun et al., 2014). Heterologous expression of Arabidopsis 14-3-3 gene *AtGF14*λ in cotton improved stress tolerance under moderate drought conditions by regulating the stomatal aperture of transgenic plants (Yan et al., 2004). In another study, AtGFψ interacted with different ACC SYNTHASE (ACS) isoforms and decreased their stability, functioning as a negative regulator of constitutive freezing tolerance and cold acclimation in Arabidopsis (Catala et al., 2014).

A number of 14-3-3 family members have been reported in different plant species; for example, 13 in Arabidopsis (Rosenquist et al., 2001), 8 in rice (Chen et al., 2006), 16 in soybean (Wang et al., 2019), 17 in tobacco (Konagaya et al., 2004), 12 in *Populus* (Tian et al., 2015), 8 in sweet orange, 15 in cassava (Chang et al., 2020), 11 in grape (Cheng et al., 2018), 17 in wheat (Shao et al., 2021), 10 in *Medicago truncatula* (Qin et al., 2016), 16 in mango (Xia et al., 2022), 18 in apple (Zuo et al., 2021), 25 in banana (Li et al., 2016), 9 in *Citrus sinensis* (Lyu et al., 2021), and 21 in *Brassica rapa* (Chandna et al., 2016). Despite the documentation of 14-3-3 genes in many plant species and their investigation under different stress conditions, little is known about this gene family in rapeseed (*Brassica napus*) and its diploid progenitor *Brassica oleracea*. Only four 14-3-3 genes have been identified in *B*. *napus* to date (Zhan et al., 2010). Rapeseed is the second most important oilseed crop in the world, and Sclerotinia stem rot (SSR), which is caused by *Sclerotinia sclerotiorum*, is a serious problem in the rapeseed industry(Wu et al., 2016). In this study, taking advantage of the completion of the newly assembled rapeseed genome sequence (Song et al., 2020), we systemically identified members of the 14-3-3 gene family in the *B*. *napus* genome, performed a comprehensive sequence analysis of these genes, and investigated their function in *S*. *sclerotiorum* resistance. Our results demonstrate that the oilseed rape genome contains the highest number of *14-3-3s* (46 *BnaGRFs*) among all plant species investigated to date, and 19 of these genes are likely involved in disease resistance, probably through the SA signaling pathway.

## Materials and methods

### Sequence search and annotation of *14-3-3* genes

Genome sequence and gene annotations of oilseed rape (*B. napus* L.) cultivar ‘Zhongshuang11’ (ZS11) were downloaded from the *B. napus* pan-genome information resource database (BnPIR^1^). The Hidden Markov Model (HMM) profiles of 14-3-3 domains (PF00244) were obtained from the Protein family database (Pfam^2^), and was employed as a query to identify all possible BnaGRFs using the HMMER software (V3.0) (Finn et al., 2011). Then, the motif of the candidate protein was confirmed using the online tools SMART,^3^ CCD,^4^ and MOTIF Search.^5^ The length, isoelectric point (pI), and molecular weight (MW) of 14-3-3 proteins were calculated using the online ExPASy program,^6^ and their subcellular localization was predicted using the online tool WOLFPSORT.^7^

The genome sequence of *B. oleracea* was downloaded from Ensembl Plants,^8^ and *B. oleracea* 14-3-3 proteins (BolGRFs) were identified using the approaches mentioned above. Additionally, 21 14-3-3 proteins (BraGRFs) in *B*. *rapa* were obtained from (Chandna et al., 2016).

### Genomic organization and synteny of *BnaGRFs* in *B. napus*

To visualize the location of *BnaGRFs* in the genome and identify syntenic gene pairs, the locations of *BnaGRFs* were extracted from the gff files of the *B*. *napus* genome.^9^ All protein sequences of *B*. *napus* were compared against themselves using the MCScanX tool of the TBtools software (*E*-value < 1e−10, number of hits ≤5) (Chen et al., 2020), and the duplicated gene groups and tandem duplicates were as described previously (Pi et al., 2021). The chromosomal locations and duplication events of *BnaGRFs* were visualized using the Circos package of the TBtools software. The ratios of non-synonymous to synonymous substitutions (Ka/Ks) in duplicate gene pairs were used to evaluate the selection pressure (Wang et al., 2010).

### Phylogenetic analysis of *14-3-3* genes

To gain insights into the evolutionary relationships among *14-3-3* family members, the structure of *BnaGRFs* and *A*. *thaliana 14-3-3s* (*AtGRFs*) was visualized using TBtools, based on the information in gff files. The conserved motifs of BnaGRFs and AtGRFs were analyzed using Multiple Expectation Maximization for Motif Elicitation (MEME) (Bailey et al., 2015), and the results were visualized with TBtools.

Amino acid sequences of the 14-3-3 proteins of *B*. *rapa*, *B*. *oleracea*, *B*. *napus*, and *A*. *thaliana* were aligned using MAFFT v7.505 with default parameters (Nakamura et al., 2018). Maximum-likelihood phylogenetic trees were generated in the MEGA 11 program using the Neighbor-Joining (NJ) method with 1,000 bootstrap replications. Additionally, phylogenetic analysis of BnaGRFs and AtGRFs was performed for investigating gene structure, while that of only BnaGRFs was performed for visualizing gene expression.

### Expression profiling of *BnaGRFs*

A highly comprehensive time-series analysis of the transcriptome in different tissues, and gene expression pattern under adverse conditions and following different hormone treatments was reported in *B*. *napus* cultivar ZS11 (Liu et al., 2021). These data were used in this research. To predict the potential functions of *BnaGRFs*, the expression patterns were illustrated using HeatMap in TBtools (Chen et al., 2020). The heatmap was constructed by taking Log2 values of the transcripts per kilobase of exon model per million mapped reads (TPM) generated from RNA-seq data. The bars of *BnaGRFs* on heatmap represent the expressional change after different treatments.

### Plant material, hormone treatments, and *S. sclerotiorum* inoculation

To probe the potential roles of *BnaGRFs* in resistance to *S. sclerotiorum*, the expression of *BnaGRFs* was inspected by RNA-seq in leaves treated with SA, ethylene (ET), hydrogen peroxide (H_2_O_2_), and oxalic acid (OA) or inoculated with *S*. *sclerotiorum*. Seed germination and seedling cultivation of *B*. *napus* cultivar ‘J9712’ were conducted in a greenhouse at 24°C under 16 h light/8 h dark photoperiod. Leaves of 6-week-old plants were sprayed with hormones (1 mM SA, 1 mM ET, or 100 mM H_2_O_2_), a chemical (1 mM OA), the toxin secreted by the pathogen *S. sclerotiorum*, or the solvent for the above compounds (0.01% ethanol; control), and collected at 0, 3, and 6 h after treatment. All samples were immediately frozen in liquid nitrogen and stored at −80°C until needed for RNA extraction (Rahman et al., 2016).

To conduct pathogen inoculation, the leaves of 6-week-old plants were treated with agar disks containing the hyphae of *S. sclerotiorum*, as described previously (Wu et al., 2013). Briefly, the latest or penultimate fully extended leaves of similar size were excised from 6-week-old plants grown in the field. Nine leaves were collected and placed in a plastic tray (56 cm × 38 cm × 15 cm) with wet-gauze at the bottom of the tray. The mycelial agar plug was inoculated on the middle of each leaf. The inoculated leaves were further sprayed with a fine mist of water, and the plastic tray was covered with plastic film to maintain a high level of relative humidity. The plastic trays with inoculated leaves were kept at 22 ± 2°C in darkness. The leaves were sampled at 0, 6, 12, 24, and 36 h after inoculation.

### RNA isolation and gene expression analysis

Leaves were collected at 0, 12, 24, and 36 h post-inoculation, with three biological replicates at each time point. Total RNA was extracted from the leaf samples using the RNA Purification Kit (TransGen, China), according to the manufacturer’s instructions. The quality of RNA was evaluated by agarose gel electrophoresis and with a UV spectrophotometer. High-quality RNA was outsourced to Majorbio company (Shanghai, China) for library construction, RNA-seq, and the identification and analysis of differentially expressed genes (DEGs). The RNA-seq was performed based on Illumina platform. The raw data was filtered with SeqPrep and Sickle, and the clean data were mapped onto the *B*. *napus* genomes (AACC^10^) (Chalhoub et al., 2014) by using TopHat2 software. The expression profiles of DEGs between the treated and control samples were determined by edgeR (Robinson et al., 2010) program based on their relative quantities. Genes with a *P*-value ≤ 0.01 were recognized as significantly DEGs between the two samples. Transcript levels of *BnaGRFs* were extracted from the RNA-seq data and shown by HeatMap in TBtools. The heatmap was generated by taking Log2 values of the transcript per million fragments mapped (FPKM) generated from RNA-seq data. The bars of *BnaGRFs* on heatmap represent the expressional change after different treatments.

## Results

### Genome-wide identification and characterization of 14-3-3 proteins in rapeseed

The genome of *B. napus* cultivar ‘ZS11’ was used for the identification of rapeseed *14-3-3* genes. Based on a genome-wide investigation, a total of 46 *14-3-3* gene candidates were discovered (Table 1). Each gene was named *BnaGRF*, followed by a number (e.g., *BnaGRF1*, *BnaGRF2*, and *BnaGRF3*), based on its location on chromosomes A01 to C09. The details of all 46 *BnaGRFs* are summarized in Table 1. The BnaGRF proteins varied in length from 182 amino acids (aa; BnaGRF28) to 581 aa (BnaGRF23), with an average length of 266.23 aa, the length of 86.95% (40/46) *BnaGRFs* distribute in 200–300 aa. The MW of BnaGRFs ranged from 20.53 to 65.58 kDa. The pI was less than 7.0 for all BnaGRFs, except BnaGRF1, BnaGRF23, and BnaGRF45, whose pI was 7.01, 9.52, and 9.66, respectively. Subcellular localization prediction analysis showed that BnaGRFs localized to different subcellular compartments, including plasma membrane (12 BnaGRFs), nuclear membrane (11), chloroplast (8), nucleus (5), cytosol (4), Golgi apparatus (3), and mitochondrion (3).

**TABLE 1:** Information on rapeseed 14-3-3 genes.

Gene ID	Gene name	Location	Exon number	Size (aa)	MW (Da)	pI	Duplication type	Subcellular localization prediction
BnaA01G0224300ZS	BnaGRF1	ChrA01:14566057:14567144(+)	3	185	20,992.32	9.52	Dispersed	Cytosol
BnaA02G0231200ZS	BnaGRF2	ChrA02:14989506:14990918(+)	4	260	29,284.76	4.61	WGD or segmental	Plasma membrane
BnaA03G0040300ZS	BnaGRF3	ChrA03:1854228:1855164(−)	2	250	28,017.69	4.83	WGD or segmental	Nucleus membrane
BnaA03G0207200ZS	BnaGRF4	ChrA03:10808510:10810092(−)	6	272	30,608.46	4.8	WGD or segmental	Nucleus membrane
BnaA03G0288100ZS	BnaGRF5	ChrA03:15284066:15285120(−)	4	261	29,439.1	4.69	WGD or segmental	Chloroplast
BnaA04G0101600ZS	BnaGRF6	ChrA04:11985269:11986295(+)	4	257	28,877.5	4.72	WGD or segmental	Nucleus membrane
BnaA05G0316100ZS	BnaGRF7	ChrA05:31427849:31428915(−)	3	263	29,670.25	4.8	WGD or segmental	Nucleus membrane
BnaA05G0489800ZS	BnaGRF8	ChrA05:44320262:44321341(+)	4	261	29,465.05	4.7	WGD or segmental	Plasma membrane
BnaA05G0493100ZS	BnaGRF9	ChrA05:44440809:44441890(−)	4	261	29,449.06	4.7	WGD or segmental	Plasma membrane
BnaA07G0108700ZS	BnaGRF10	ChrA07:15374454:15375753(−)	6	245	27,976.12	4.72	WGD or segmental	Nucleus
BnaA07G0123200ZS	BnaGRF11	ChrA07:16439637:16440852(−)	4	260	29,156.83	4.7	WGD or segmental	Plasma membrane
BnaA07G0123900ZS	BnaGRF12	ChrA07:16484239:16485700(+)	6	253	28,752.22	4.76	WGD or segmental	Nucleus
BnaA07G0167800ZS	BnaGRF13	ChrA07:19568278:19569285(+)	4	258	28,964.53	4.74	WGD or segmental	Nucleus membrane
BnaA07G0235600ZS	BnaGRF14	ChrA07:23323067:23324443(−)	4	260	29,198.71	4.65	WGD or segmental	Plasma membrane
BnaA07G0373400ZS	BnaGRF15	ChrA07:31468756:31469982(−)	5	212	24,186.73	6.65	WGD or segmental	Cytosol
BnaA07G0374100ZS	BnaGRF16	ChrA07:31504845:31506571(+)	3	299	33,657.87	4.92	WGD or segmental	Chloroplast
BnaA08G0230100ZS	BnaGRF17	ChrA08:24101625:24103213(−)	7	328	37,702.26	4.89	WGD or segmental	Golgi apparatus
BnaA09G0091600ZS	BnaGRF18	ChrA09:5340343:5341619(−)	3	249	28,066.8	4.82	WGD or segmental	Nucleus membrane
BnaA09G0375500ZS	BnaGRF19	ChrA09:43270421:43271551(−)	4	261	29,793.4	4.75	WGD or segmental	Chloroplast
BnaA09G0441400ZS	BnaGRF20	ChrA09:49887816:49889023(−)	6	269	30,599.21	4.77	WGD or segmental	Nucleus membrane
BnaA09G0462600ZS	BnaGRF21	ChrA09:51319481:51321117(+)	6	253	28,752.21	4.74	WGD or segmental	Mitochondrion
BnaA10G0242300ZS	BnaGRF22	ChrA10:23807919:23808830(+)	2	248	27,991.74	4.72	WGD or segmental	Chloroplast
BnaC01G0287900ZS	BnaGRF23	ChrC01:25148028:25151640(+)	14	581	65,584.35	7.01	WGD or segmental	Nucleus
BnaC02G0311500ZS	BnaGRF24	ChrC02:30481661:30483110(+)	4	260	29,284.76	4.61	WGD or segmental	Plasma membrane
BnaC03G0244000ZS	BnaGRF25	ChrC03:14931129:14932753(−)	6	271	30,495.34	4.77	WGD or segmental	Nucleus membrane
BnaC03G0346100ZS	BnaGRF26	ChrC03:23526445:23527500(−)	4	261	29,409.07	4.7	WGD or segmental	Plasma membrane
BnaC03G0626500ZS	BnaGRF27	ChrC03:60205248:60206819(+)	7	328	37,691.27	4.89	WGD or segmental	Golgi apparatus
BnaC04G0029800ZS	BnaGRF28	ChrC04:2882891:2883539(+)	2	182	20,530.78	4.62	Dispersed	mitochondrion
BnaC04G0380200ZS	BnaGRF29	ChrC04:50440648:50441711(−)	4	257	28,847.51	4.74	WGD or segmental	Chloroplast
BnaC05G0194000ZS	BnaGRF30	ChrC05:13641970:13643756(−)	6	253	28,780.22	4.74	WGD or segmental	Mitochondrion
BnaC05G0221900ZS	BnaGRF31	ChrC05:16739710:16740903(+)	6	270	30,708.38	4.81	WGD or segmental	Nucleus membrane
BnaC05G0329400ZS	BnaGRF32	ChrC05:35401996:35403560(−)	4	265	30,023.66	4.76	WGD or segmental	Plasma membrane
BnaC06G0098700ZS	BnaGRF33	ChrC06:17625723:17627380(+)	7	394	45,018.09	5.61	Dispersed	Cytosol
BnaC06G0157000ZS	BnaGRF34	ChrC06:25447602:25448659(+)	4	257	28,891.48	4.74	WGD or segmental	Nucleus membrane
BnaC06G0256100ZS	BnaGRF35	ChrC06:36385398:36386709(−)	4	260	29,198.71	4.65	WGD or segmental	Plasma membrane
BnaC06G0439100ZS	BnaGRF36	ChrC06:51346480:51347852(−)	5	241	27,385.18	5.31	WGD or segmental	Cytosol
BnaC06G0440000ZS	BnaGRF37	ChrC06:51399614:51401340(+)	4	260	29,226.72	4.64	WGD or segmental	Plasma membrane
BnaC07G0163800ZS	BnaGRF38	ChrC07:29220090:29221461(−)	7	267	30,723.14	4.75	WGD or segmental	Nucleus
BnaC07G0181300ZS	BnaGRF39	ChrC07:31130202:31131472(−)	4	260	29,142.85	4.74	WGD or segmental	Plasma membrane
BnaC07G0181600ZS	BnaGRF40	ChrC07:31157168:31158438(−)	4	260	29,142.85	4.74	Proximal	Plasma membrane
BnaC07G0182400ZS	BnaGRF41	ChrC07:31284178:31285716(+)	7	253	28,779.25	4.76	WGD or segmental	Nucleus
BnaC08G0272000ZS	BnaGRF42	ChrC08:35792611:35793759(−)	5	269	30,756.99	5.23	WGD or segmental	Golgi apparatus
BnaC08G0272400ZS	BnaGRF43	ChrC08:35866753:35867833(+)	4	259	29,002.58	4.63	WGD or segmental	Chloroplast
BnaC09G0088500ZS	BnaGRF44	ChrC09:5828367:5830224(−)	3	249	28,048.77	4.82	WGD or segmental	Chloroplast
BnaC09G0404100ZS	BnaGRF45	ChrC09:51468318:51470346(−)	4	197	22,690.4	9.66	Dispersed	Chloroplast
BnaC09G0553200ZS	BnaGRF46	ChrC09:64088276:64093727(+)	3	228	25,371.85	4.81	WGD or segmental	Nucleus membrane

To better understand the *14-3-3* family composition in the tetraploid *B*. *napus*, the *14-3-3* genes in its progenitor species *B*. *rapa* (*BraGRFs*) and *B*. *oleracea* (*BolGRFs*) were identified; *BolGRFs* were identified using approaches similar to those used for *BnaGRF* identification, while *BraGRFs* were identified based on the literature reported by Chandna (Chandna et al., 2016). The results showed that the *14-3-3* gene family composition was similar in the two *Brassica* species, with 21 members in *B*. *rapa* and 25 in *B*. *oleracea* (Supplementary Table 1). Comparative analysis indicated that the *B*. *napus* genome possessed the total copies of *14-3-3s* in its two progenitor species, but the A and C subgenomes possessed 22 and 24 *BnaGRFs*, respectively.

### Phylogenetic and gene structure analyses of *14-3-3* genes

**FIGURE 1:**
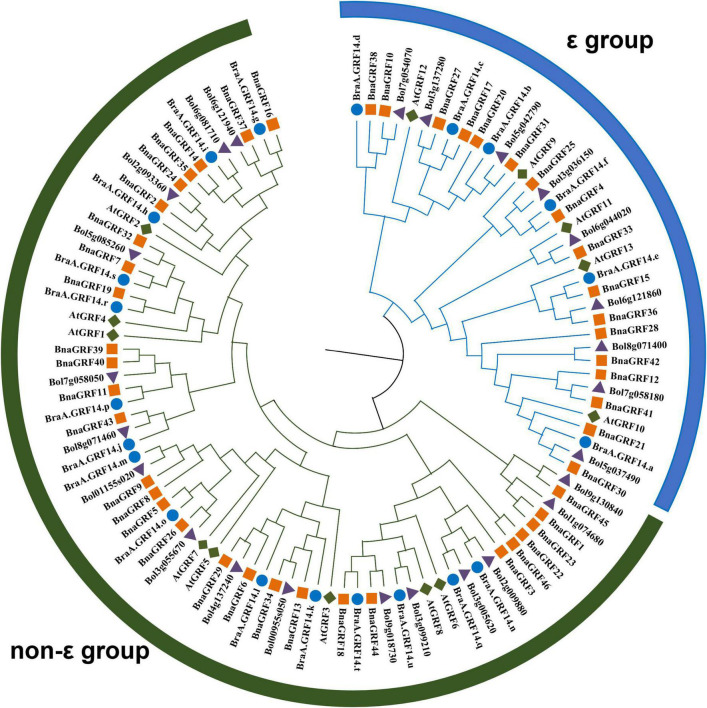
Phylogenetic analysis of the 14-3-3 protein family in *Brassica* species (*B*. *rapa*, *B*. *oleracea*, and *B*. *napus*) and *Arabidopsis thaliana*. A total of 46 14-3-3s were divided into two subgroups (ε and non-ε group), according to the evolutionary distance. Blue circles represent the 21 BraGRFs in *B*. *rapa*; purple triangles represent the 25 BolGRFs in *B*. *oleracea*; orange squares represent the 46 BnaGRFs in *B*. *napus*; and green diamonds represent the 13 AtGRFs in Arabidopsis.

To determine the evolutionary relationships among *14-3-3s*, a phylogenetic tree was constructed using the NJ method, based on the full-length amino acid sequence alignment of GRFs of *B*. *rapa*, *B*. *oleracea*, *B*. *napus*, and *A*. *thaliana* (Figure 1). All 46 BnaGRFs clustered into two groups (ε and non-ε), with strong bootstrap support. Twenty-nine BnaGRFs clustered into the non-ε group, together with 8 AtGRFs, 15 BraGRFs, and 16 *B*. *oleracea* GRFs (BolGRFs), while the remaining 17 BnaGRFs members clustered into the ε group. *B*. *napus* inherited most copies of *14-3-3s* from its two progenitors, *B*. *rapa* and *B*. *oleracea*. Some BnaGRFs were highly homologous to BolGRFs; e.g., both BnaGRF12 and BnaGRF41. However, other BnaGRFs, such as BnaGRF22 and BnaGRF46, showed no high-level homology to GRFs in *B*. *rapa* and *B*. *oleracea*. Thus, this phylogenetic tree revealed that members of the *14-3-3* gene family in *B*. *napus* were not fully inherited from its diploid ancestors.

**FIGURE 2:**
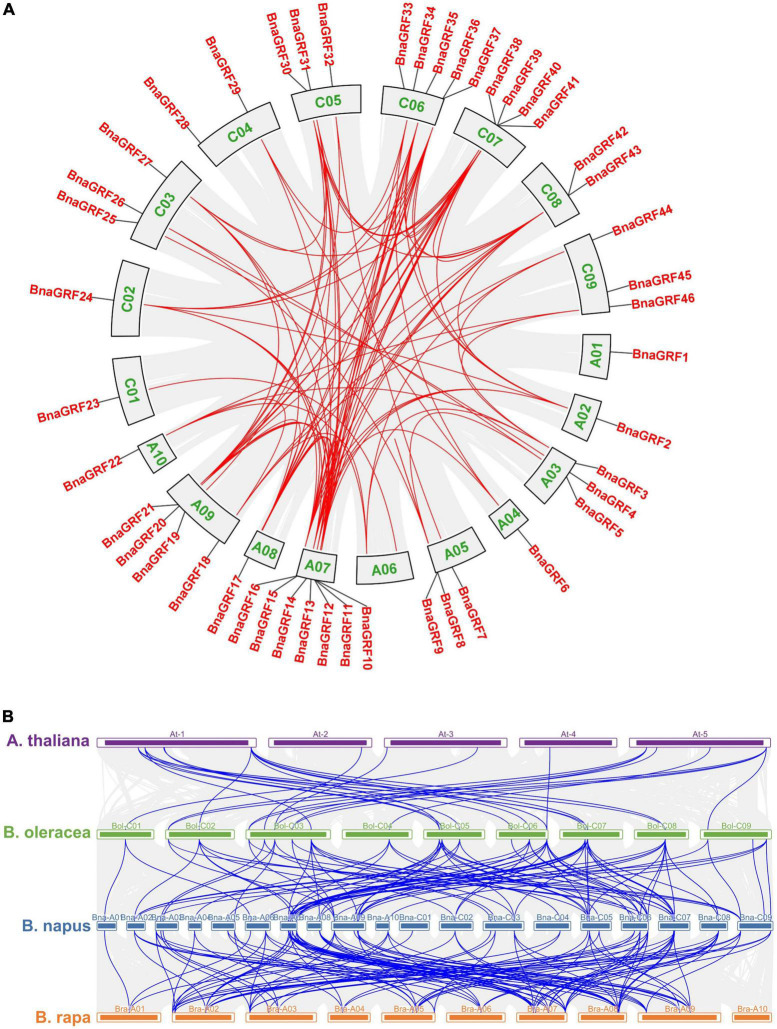
Circular illustrations of the chromosomal dispersal and inter-chromosomal associations of BnaGRFs, and synteny analysis of *14-3-3* genes in *B. rapa*, *B. oleracea*, *B. napus*, and Arabidopsis. **(A)** Analysis of syntenic relationships among BnaGRF homologs. Red lines inside the schematic view denote the duplicated pairs of BnaGRFs. **(B)** Analysis of syntenic relationships among BraGRFs, BolGRFs, BnaGRFs, and AtGRFs. Gray line represents the syntenic block in plant genomes, and blue line represents the collinear *14-3-3* gene pair.

The intron-exon structure of *14-3-3s* provided potential insight into the functional diversification of BnaGRFs during evolution (Table 1 and Figure 2A). Genes within the same group showed a similar number of introns and exons: 2–4 exons in the non-ε group [with 20 out of 28 genes (68.96%) containing 4 exons] and 5–7 exons in the ε group. The only exceptions were *BnaGRF23* in the non-ε group (14 exons) and *BnaGRF28* in the ε group (2 exons).

Ten conserved motifs were identified in 46 BnaGRFs and 13 AtGRFs (Figure 2B). The majority of BnaGRFs contained motif2, motif3, and motif4, except BnaGRF1, BnaGRF28, BnaGRF45 (which lacked motif2 and motif4), BnaGRF23 (lacked motif2), BnaGRF42 (lacked motif3), and BnaGRF46 (lacked motif4). Most BnaGRFs (40/46) contained motif1, except BnaGRF3, BnaGRF22, and BnaGRF46, all of which clustered in the non-ε group. Motif5 was found in most non-ε group members (21/29), whereas motif8 and motif9 were found in ε group members only. By contrast, AtGRFs showed a more consistent motif distribution than BnaGRFs, with all AtGRFs containing motif1, motif2, motif3, and motif4. Compared with all BnaGRFs, the non-ε group members BnaGRF1, BnaGRF3, BnaGRF22, BnaGRF23, BnaGRF45, and BnaGRF46 as well as the ε group member BnaGRF28 showed considerable differences in intron-exon structure, motif distribution, MW, and pI (Table 1). This variation in gene structure and motif composition among BnaGRFs suggests functional differentiation within and between the two BnaGRFs groups.

### Chromosomal distribution and gene duplication of *BnaGRFs*

**FIGURE 3:**
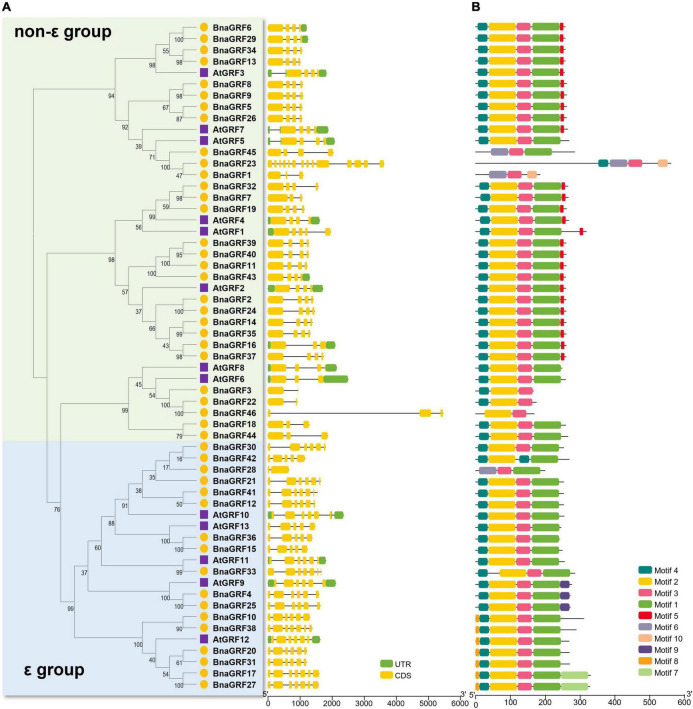
Gene structure and motif analysis of *14-3-3* family genes in *B. napus* and Arabidopsis. **(A)** Gene structure of *BnaGRFs* and *AtGRFs*. Green color indicates the untranslated regions (UTRs); yellow color represents the coding sequence (CDS) or exons; and black horizontal lines indicate the introns. **(B)** Conserved domain structures identified in *BnaGRFs* and *AtGRFs*. Boxes with different colors indicate different motifs.

The 46 *BnaGRF* genes were unevenly distributed on 18 of the 19 chromosomes (Figure 3). In total, 22 *BnaGRFs* (15 non-ε group and 7 ε group) and 24 *BnaGRFs* (14 non-ε group and 10 ε group) were located in the A and C subgenomes, respectively. Chromosome A07 harbored the most *BnaGRF* genes (seven), while chromosomes A01, A02, A04, A08, A10, C01, and C02 contained only one *BnaGRF* gene each. No *BnaGRF* gene was located on chromosome A06.

**FIGURE 4:**
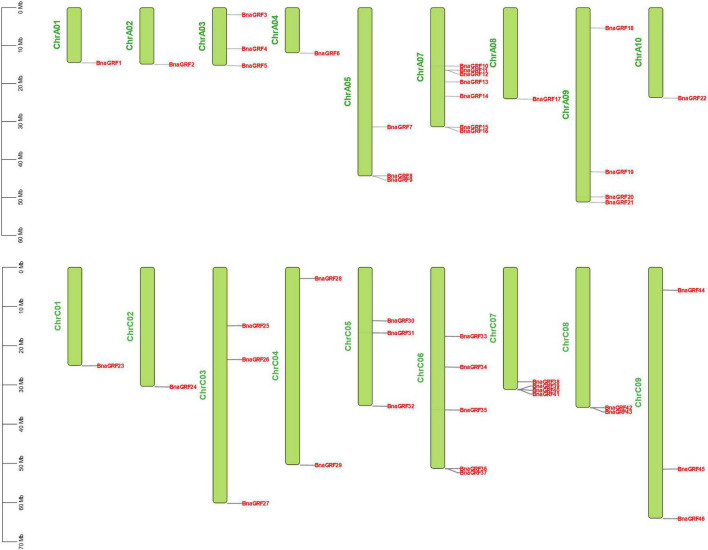
Chromosomal mapping of 46 *BnaGRFs*. The length of chromosomes is represented in Mb. The chromosome number is presented on the left side of each chromosome. *BnaGRFs* are marked in red.

Gene duplication reflects the expansion of the gene family. According to BLAST and MCScanX searches, gene duplication events of *BnaGRFs* were detected in *B*. *napus*. Briefly, all 46 *BnaGRF* genes were derived by different types of duplication events: 41 (89.13%) by whole-genome duplication (WGD) or segmental duplication; 4 by dispersed duplication; and 1 by proximal duplication. Moreover, we identified 101 paralogous gene pairs (Figure 2A and Supplementary Table 2), 24 of which originated in the A subgenome, 18 in the C subgenome, and the remaining 59 arose by duplication events between the A and C subgenomes. To estimate the selection mode of *BnaGRFs*, the Ka/Ks ratio of paralogous gene pairs was calculated. Generally, Ka/Ks > 1 implies positive selection; Ka/Ks = 1 indicates neutral selection; and Ka/Ks < 1 represents purifying selection. In this study, the Ka/Ks ratio of all paralogous gene pairs (except three gene pairs for which the Ka/Ks ratio could not be calculated, because of high sequence divergence) was less than 1 (Supplementary Table 2), indicating that *BnaGRFs* are under purifying selection.

### Prediction of *Cis*-acting elements in *BnaGRF* gene promoters

**FIGURE 5:**
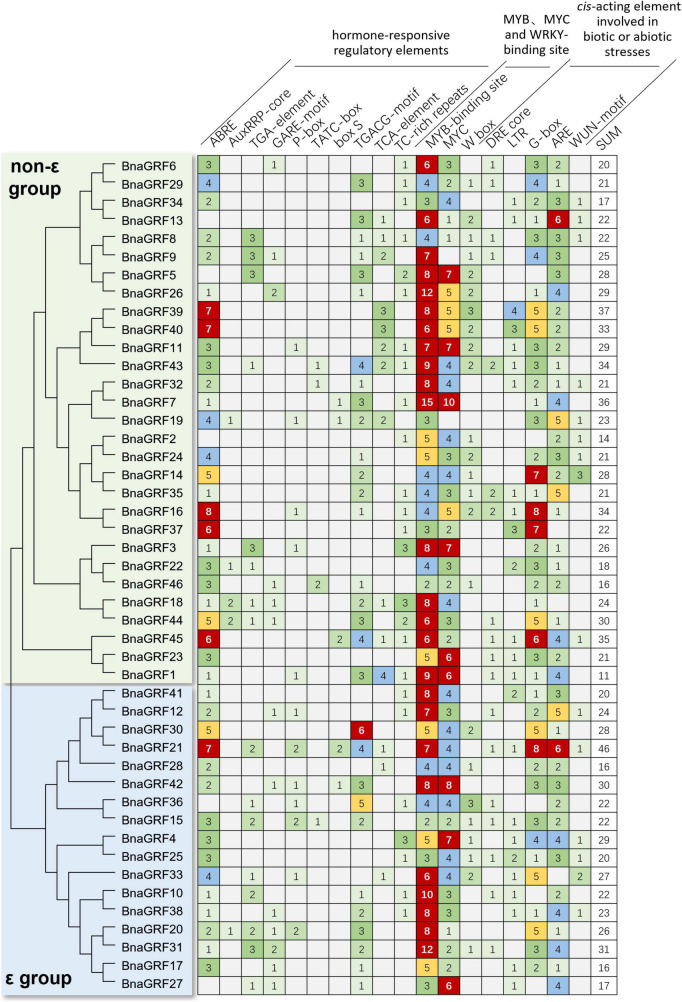
Number of hormone- and stress-responsive *cis*-elements in the promoters of the *BnaGRFs*.

*Cis*-elements in promoter regions play a critical role in determining gene expression patterns. To obtain preliminary clues on how the *BnaGRF* genes respond to stress stimuli, we identified stress-related *cis*-elements in the promoters of *BnaGRF* genes (Figure 5). A total of 18 stress response-related *cis*-acting elements were detected in 2.0-kb sequence upstream of the ATG of each *BnaGRF* gene. These *cis*-acting elements could be divided into three classes: hormone-sensitive regulatory elements involved in biotic or abiotic stress responses, including ABA-responsive element (ABRE), indole-3-acetic acid (IAA)-responsive elements (AuxRRP-core and TGA-element), GA-responsive elements (GARE-motif, P-box, and TATC-box), JA responsive (TGACG-motif and box S), and SA-responsive element (TCA-element); MYB, MYC, and WRKY transcription factor-binding sites; and biotic or abiotic stress-responsive elements, including DRE core, LTR, G-box, ARE, TC-rich repeats, and WUN-motif. MYB-binding elements were detected in all 46 *BnaGRFs*, with a total number of 284. Other motifs identified in the majority of *BnaGRFs* included ABRE (41/46, 89.1%), MYC-binding (44/46, 95.7%), G-box (41/46, 89.1%), and ARE (43/46, 93.5%). More than half of *BnaGRFs* promoters contained methyl jasmonate (MeJA)-responsive elements [TGACG-motif (30/46, 65.2%) and TC-rich repeats (25/46, 54.3%)], WRKY-binding site (W box, 24/46, 52.2%) and low-temperature responsive (LTR) elements (24/46, 52.2%). Moreover, TGA-element (16/46, 34.8%), GARE-motif (13/46, 28.3%), TCA-element (14/46, 30.4%), DRE core (18/46, 39.1%), and WUN-motif (15/46, 32.6%) were identified in many promoters. The total number of elements in each gene varied from 14 (*BnaGRF2*) to 46 (*BnaGRF21*), and no significant difference was detected in the number or distribution of elements between non-ε and ε groups. These findings suggest that BnaGRFs play an important role in the biotic and abiotic stress response in *B*. *napus*.

### Expression profiling of *BnaGRF* genes in various *B. napus* tissues

**FIGURE 6:**
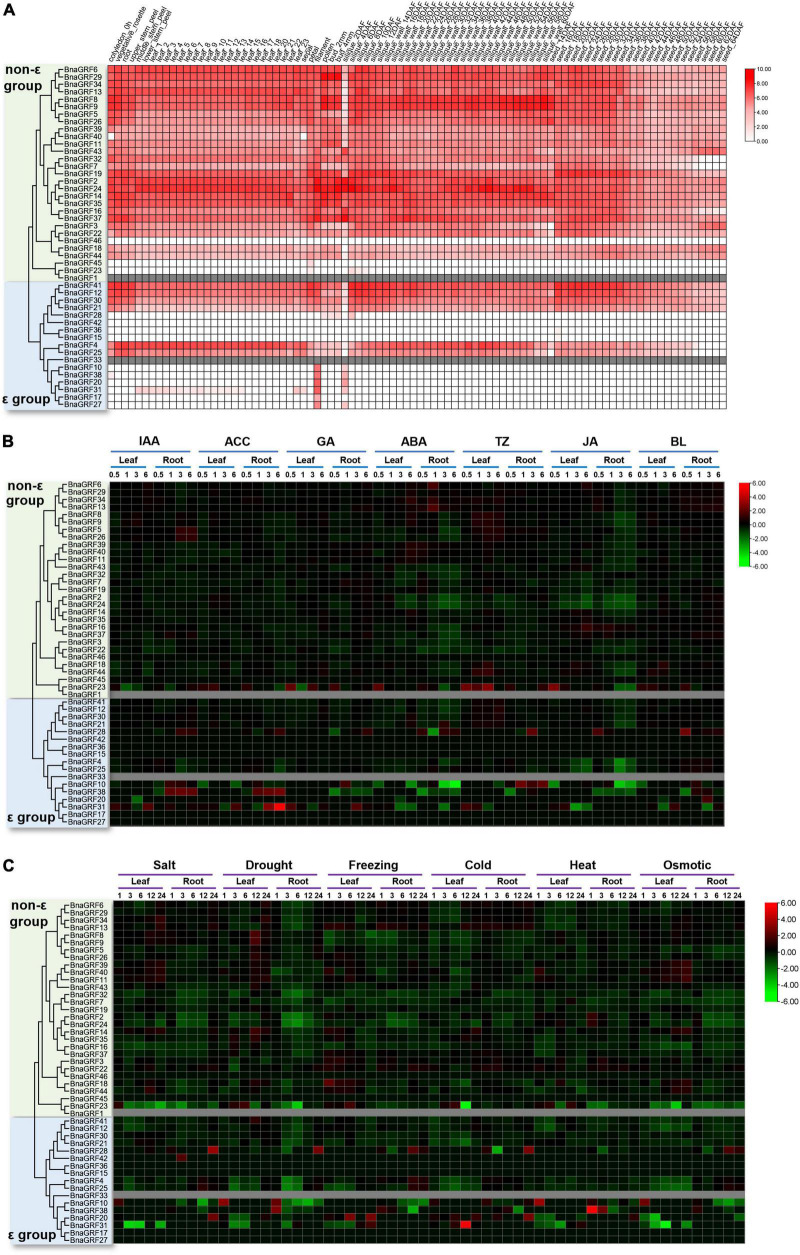
Expression analysis of *BnaGRFs* in various tissues and under various hormone and abiotic stress treatments. **(A)** Expression profiling in different tissues and organs. The scale bar represents the Log2 normalized expression. In the expression bar, white color indicates no expression, and gray color indicates the absence of data in the BnTIR database (*BnaGRF1* and *BnaGRF33*). **(B)** Expression profiles of *BnaGRFs* after treatment with various hormones, including indole-3-acetic acid (IAA), 1-aminocyclopropane-1-carboxylic acid (ACC), gibberellin (GA), ABA, trans-Zeatin (TZ), jasmonate (JA), and brassinolide (BL). The heatmap was constructed by taking Log2 values of the transcripts per kilobase of exon model per million mapped reads (TPM) generated from RNA-seq data. The bars of *BnaGRFs* on heatmap represent the expression change folds after treatments. **(C)** Expression profiles of *BnaGRFs* under abiotic stresses, including salt (200 mM NaCl), drought, freezing (–4°C), cold (4°C), heat (38°C), and osmotic stress (300 mM mannitol). In panels **(B,C)**, red and green colors indicate up- and downregulated *BnaGRFs*, respectively, and gray color indicates unavailability of data in the database (*BnaGRF1* and *BnaGRF33*). The heatmap was constructed by taking Log2 values of the transcripts per kilobase of exon model per million mapped reads (TPM) generated from RNA-seq data. The bars of *BnaGRFs* on heatmap represent the expression change folds after treatments.

To investigate the functions of *BnaGRF* genes, we analyzed their expression profiles in different tissues or organs based on the data available in the *Brassica napus* Transcriptome Information Resource (BnTIR) database^11^ (Liu et al., 2021). Different *BnaGRF* genes exhibited distinct expression patterns; however, no transcriptome data were available for BnaGRF1 and BnaGRF33 (Figure 6A).

Most *BnaGRFs* were widely expressed in the *B*. *napus* plant at different developmental stages. However, some *BnaGRFs*, including *BnaGRF15*, *BnaGRF23*, *BnaGRF28*, *BnaGRF36*, *BnaGRF42*, *BnaGRF45*, and *BnaGRF46*, showed no expression in most of the tissues and organs; three of these genes belonged to the non-ε group, while four of them belonged to the ε group. *BnaGRF8* and *BnaGRF9* were highly expressed in the silique wall at 60 days after flowering (DAF), suggesting that both these genes play specific roles in silique dehiscence in *B*. *napus*. Additionally, *BnaGRF10*, *BnaGRF17*, *BnaGRF20*, *BnaGRF27*, and *BnaGRF38*, all of which belonged to the ε group, were expressed only in the filament and 2-DAF silique. Interestingly, many *BnaGRFs* were expressed to significantly lower levels in siliques at 2 DAF than in siliques at other developmental stages. These results suggest that *BnaGRFs* play important roles in the growth and development of *B*. *napus*.

### Responses of *BnaGRFs* to phytohormones, abiotic stress, and *S. sclerotiorum* challenge

The expression profiles of *BnaGRFs* were analyzed in different tissues after treatment with various hormones [IAA, 1-aminocyclopropane-1-carboxylic acid (ACC), GA, ABA, trans-Zeatin (TZ), JA, and BL] and abiotic stresses (salt, drought, freezing, cold, heat, and osmotic), based on the data available in BnTIR (Figures 6B,C).

Heatmap analysis showed that the *BnaGRF* genes were downregulated upon most hormone treatments (Figure 6B), and were upregulated only in a few instances; however, six genes (*BnaGRF15*, *BnaGRF17*, *BnaGRF27, BnaGRF36*, *BnaGRF45*, and *BnaGRF46*) were either not expressed or expressed to very low levels in all hormone treatments. Most *BnaGRFs* were downregulated after ABA and JA treatments; the expression of a few genes either increased or did not change significantly. *BnaGRFs* showed different expression patterns in roots and leaves. For instance, *BnaGRF10* and *BnaGRF38* were down- or upregulated significantly under all of the hormone treatments in the root; however, their expression changed only at a few time points. In contrast to leaves, the expression levels of *BnaGRFs* changed more dramatically in roots. These results indicate *BnaGRFs* play important roles in the response to various stresses in the root. Members of the non-ε and ε groups showed different expression patterns. Most of the non-ε group members were downregulated upon hormone treatments, except BnaGRF23, which was upregulated by all phytohormones at some point in time. By contrast, the expression levels of ε group members, including *BnaGRF10*, *BnaGRF28*, *BnaGRF31*, and *BnaGRF38* changed more dramatically. In the root, the transcript level of *BnaGRF10* decreased by 32-fold at 6 h after the ACC treatment, while that of *BnaGRF31* decreased by 42-fold at 6 h post-ABA treatment. The ε group members were more frequently upregulated than the non-ε group members. For example, the transcript level of *BnaGRF28* was increased in the root at one or more sampling points after all hormone treatments; *BnaGRF10*, *BnaGRF31*, and *BnaGRF38* were significantly upregulated at some sampling points after treatment with IAA, ACC, GA, TZ, and BL. Additionally, paralogous gene pairs, such as *BnaGRF2*/*BnaGRF24*, *BnaGRF18*/*BnaGRF44*, and *BnaGRF4*/*BnaGRF25*, showed similar expression patterns.

Under abiotic stress conditions, most of the *BnaGRFs* showed relatively low expression levels, particularly in roots. All BnaGRFs were downregulated in roots under salt and drought stress, except *BnaGRF10*, *BnaGRF20*, *BnaGRF28*, and *BnaGRF42*, which were upregulated. Members of both non-ε and ε groups showed similar expression patterns under abiotic stresses ass under hormone treatments. Compared with the ε group members, the non-ε group members showed a smooth transition in expression levels from pre-treatment to post-treatment conditions, except *BnaGRF23*, which was downregulated by 31.00-fold in leaves at 12 h after under the cold stress treatment; *BnaGRF38*, which was significantly upregulated by 47-fold in roots at 1 h after the heat treatment; and *BnaGRF31*, which was upregulated by 43-fold and downregulated by 32-fold at 12 h after cold treatment and 6 h after osmotic stress treatment, respectively. These results indicate that *BnaGRFs* perform biologically relevant functions during plant development and are potentially involved in abiotic stress responses.

**FIGURE 7:**
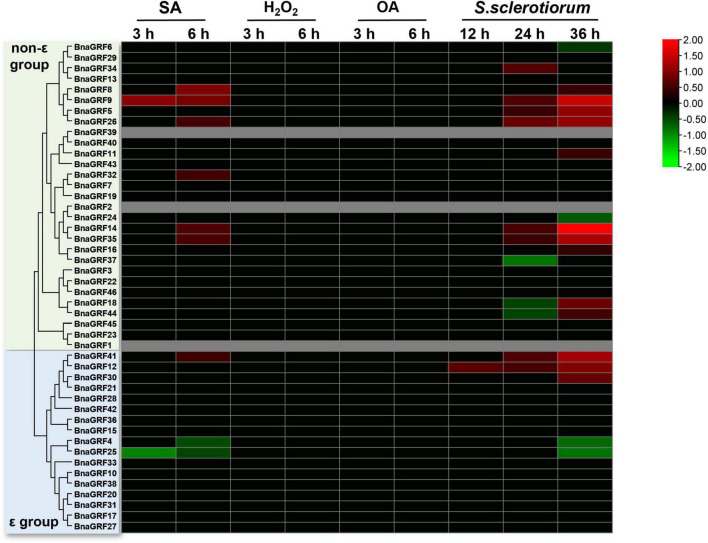
Relative expression levels of *BnaGRFs* under salicylic acid (SA), hydrogen peroxide (H_2_O_2_), and oxalic acid (OA) treatments and *Sclerotinia sclerotiorum* inoculation. No significant change in expression (*p* > 0.01) is shown as 0. Red and green colors indicate significantly up- and downregulated genes, respectively, and gray color represents no expression data. The heatmap was constructed by taking Log2 values of the transcript per million fragments mapped (FPKM) generated from RNA-seq data. The bars of *BnaGRFs* on heatmap represent the expression change folds after treatments.

Next, to investigate the role of *BnaGRFs* in the disease resistance response, the expression levels of these genes were determined by RNA-seq analysis of leaves treated with SA, H_2_O_2_, and OA or inoculated with *S. sclerotiorum*. The DEGs were identified after treatments at different time points (Supplementary Figure 1). SA and H_2_O_2_ are important signaling molecules, and OA is a main pathogenic factor secreted by *S*. *sclerotiorum*. Our results showed that no *BnaGRFs* were induced or repressed by H_2_O_2_ and OA (Figure 7). In the SA treatment, seven *BnaGRF* genes (*BnaGRF8*, *BnaGRF9*, *BnaGRF26*, *BnaGRF32*, *BnaGRF35*, *BnaGRF41*, and *BnaGRF14*) were upregulated, and two *BnaGRFs* (*BnaGRF4* and *BnaGRF25*) were downregulated significantly. All of these genes were induced or depressed at a late time point (6 h post-treatment). Interestingly, all of these nine *BnaGRFs* showed the same expression patterns after *S*. *sclerotiorum* inoculation, except *BnaGFR32*, which was upregulated by SA but showed no significant expression variation after *S*. *sclerotiorum* inoculation. Nineteen *BnaGRFs* were differentially expressed by *S*. *sclerotiorum* inoculation at different time points. Among these 19 genes, 12 *BnaGRFs* and 5 *BnaGRFs* were up- and downregulated, respectively, and 2 *BnaGRFs* (*BnaGRF18* and *BnaGRF44*) were downregulated at 24 h post-inoculation (hpi) but upregulated at 36 hpi.

Overall, the expression profiles of *BnaGRFs* in response to phytohormones, abiotic stresses, and *S*. *sclerotiorum* inoculation suggest that these genes perform diverse or conserved biological functions during plant–environment and plant–microbe interactions.

## Discussion

The 14-3-3 family has been found in all eukaryotes screened to date, and usually consists of multiple proteins and protein isoforms (DeLille et al., 2001). The 14-3-3 proteins exist as homo- and heterodimers in cells (Jones et al., 1995), and function as phosphor-binding regulators in signal transduction pathways by interacting with phosphorylated targets (Camoni et al., 2018). Because 14-3-3s exist as multiple isoforms, these proteins participate in various physiological processes. To the best of our knowledge, the largest *14-3-3* gene family reported to date in plants was identified in both banana and cotton, with each containing 25 *14-3-3s* (Sun et al., 2011; Li et al., 2016). However, in this research, we identified 46 *BnaGRF* genes in the *B*. *napus* genome; this number is almost twice that in banana and cotton. The lengths of BnaGRF isoforms varied from 182 to 581 aa, and their pI ranged from 4.61 to 5.61, except BnaGRF1 (9.52), BnaGRF15 (6.65), BnaGRF23 (7.01) and BnaGRF45 (9.66) (Table 1). Overall, the data on gene structure, amino acid number, motif type and number, and pI indicated that BnaGRF1, BnaGRF23, and BnaGRF45 are much different from the standard 14-3-3 isoforms, and there are no orthologous genes in Arabidopsis (Figure 1), indicating that BnaGRFs have undergone neofunctionalization in *B*. *napus*.

Phylogenetic analysis showed that *BnaGRFs* could be classified into two groups (ε and non-ε) (Figure 1), consistent with the 14-3-3 family in other plant species (Ferl et al., 2002). The ε and non-ε group members showed considerable differences in intron-exon structure and motif distribution. For instance, *BnaGRFs* in the ε group contained 5–7 exons, whereas those in the non-ε group contained 2–4 exons, except *BnaGRF28* and *BnaGRF23*, which contained 2 and 14 exons, respectively. The expression patterns of *BnaGRF28* and *BnaGRF23* under hormone treatments differed from those of other *BnaGRFs* (Figure 6). Similar results have been reported in other plant species (Cheng et al., 2018; Wang et al., 2019; Shao et al., 2021), which imply evolutionary conservation within the 14-3-3 family.

Tandem duplication, segmental duplication, and WGD events were identified to evaluate the expansion of the *BnaGRF* gene family. Our results showed that 41 *BnaGRFs* were generated by WGD or segmental duplication; four *BnaGRFs* (*BnaGRF28*, *BnaGRF23*, *BnaGRF28*, and *BnaGRF23*) were a result of dispersed duplication; *BnaGRF40*, which is separated from *BnaGRF39* by only a few genes, originated by proximal duplication; and no *BnaGRFs* were caused by tandemly duplication (Table 1 and Figure 4). These results indicate that WGD and segmental duplications were the key factors responsible for the expansion of the *14-3-3* gene family, which is consistent with previous studies (Xie et al., 2022).

*BnaGRF* genes in the A and C subgenomes of *B*. *napus* are closely related to *14-3-3* genes in *B*. *rapa* and *B*. *oleracea*, respectively. The number of *14-3-3* genes in *B*. *napus* is exactly equal to the sum of *14-3-3* genes in *B*. *rapa* and *B*. *oleracea*; however, the *14-3-3* genes in *B*. *napus* do not show a one-to-one correspondence with those in *B*. *rapa* and *B*. *oleracea*. We identified 22 and 24 *BnaGRFs* in the A and C subgenomes, respectively, of *B*. *napus*, and 21 *BraGRFs* and 25 *BolGRFs* in *B*. *rapa* and *B*. *oleracea* genomes, respectively. *BnaGRF8* (BnaA05G0489800ZS) is located on chromosome A05 and was identified as an ortholog of Bol01155s020; however, no orthologs of Bol3g099210 and Bol3g005620 were identified in the *B*. *napus* genome, even though both *BolGRFs* are highly homologous to *BnaGRF44* (BnaC09G0088500ZS). These results suggest that gene loss events likely occurred in the *14-3-3* gene family in *B*. *napus* after hybridization.

*Cis*-regulatory elements play an important role in the regulation of plant gene expression. The *cis*-acting element and expression pattern analysis showed that 14-3-3s were involved in hormone and abiotic stress responses in plants (Lyu et al., 2021; Xia et al., 2022; Xie et al., 2022). In this study, we analyzed the stress response-related *cis*-acting elements in the promoters of *BnaGRFs*. The 25 *cis*-regulatory elements were found in the majority of promoters (Figure 5), which implies that the corresponding genes are involved in the stress response.

Expression patterns of *BnaGRFs* under multiple hormone and abiotic stress treatments were also investigated in this study. Yang Lab developed the BnTIR database, presenting a highly comprehensive temporal-resolution analysis of the transcriptomes of nine different ‘ZS11’ tissues (cotyledon, root, stem peel, leaf, bud, flower, silique, silique wall, and seed) at three distinct developmental stages, and the transcriptomes of roots and leaves under multi-hormone and abiotic stress treatments (Liu et al., 2021). Spatial and temporal expression profiles showed that most of the *BnaGRFs* were expressed in the vast majority of tissues, except *BnaGRF15*, *BnaGRF23*, *BnaGRF28*, *BnaGRF36*, *BnaGRF42*, *BnaGRF45*, and *BnaGRF46*, which showed no expression in almost all tissues or organs. These results suggest that *BnaGRFs* play important roles in the regulation of plant growth and development in *B*. *napus*. Interestingly, the expression of *BnaGRF10*, *BnaGRF17*, *BnaGRF20*, *BnaGRF27*, and *BnaGRF38* was only detected in filament and silique sampled at 2 DAF; these genes were clustered into the ε group, which indicates that these genes function in floral organ development.

Phytohormones play a central role in the defense against environmental stress. Expression analysis showed most *BnaGRFs* responded to hormone and abiotic stress treatments, although the response was stronger to abiotic stresses than to hormones (Figures 6B,C). Compared with other hormones, ABA and JA treatment had a greater influence on the expression level of *BnaGRFs*. Because ABA and JA are closely related to abiotic and biotic stresses, other results hint at the involvement of *BnaGRFs* in the response to abiotic and biotic stresses. Almost all *BnaGRFs* responded to salt, drought, freezing, cold, heat, and osmotic stresses. In most cases, *BnaGRFs* were downregulated by these abiotic stresses. Expression levels of *BnaGRFs* differed significantly between the ε and non-ε groups; for instance, a higher percentage of *BnaGRFs* in the ε group was unexpressed or showed low-level expression in response to all abiotic stresses (5/17), whereas only two *BnaGRFs* in the non-ε group (*BnaGRF45* and *BnaGRF46*) showed this expression pattern. Compared with the non-ε group *BnaGRFs*, the ε group members showed a more dramatic increase or decrease in transcript levels. These results suggest that *BnaGRFs*, especially the ε group members, are extensively involved in the stress response in *B*. *napus*, and this involvement most likely occurs through various hormone signaling pathways.

Sclerotinia stem rot, caused by *S*. *sclerotiorum*, is the most serious disease affecting the yield of rapeseed, an agriculturally and economically important crop (Boland and Hall, 1994; Liu et al., 2018). JA, SA, and H_2_O_2_ signaling pathways are thought to be central components of the mechanisms underlying the active defense response (Hu et al., 2021; Xu et al., 2021). OA is a toxic compound secreted by *S*. *sclerotiorum*, which plays an important role in the infection process (Magro et al., 1984; Liang et al., 2015). In the current study, we performed SA, H_2_O_2_, and OA treatments and *S*. *sclerotiorum* inoculation to evaluate changes in the expression levels of *BnaGRFs*. None of the *BnaGRFs* showed a response to H_2_O_2_ and OA treatments; however, 9 (19.57%) and 19 (41.30%) *BnaGRFs* showed differential expression upon the SA treatment and *S*. *sclerotiorum* inoculation, respectively (Figure 7). These results indicated that *BnaGRF* family may not participate in H_2_O_2_ and OA metabolic and accumulation processes in *B*. *napus*. In addition, changes in the expression levels of *BnaGRFs* were also detected upon the JA treatment (Figure 6B). The *cis*-element analysis of these gene promoters revealed that all of the 19 *BnaGRFs* promoters have MYB-binding sites. In addition, 18 of them show MYC and G-box element and 17 have ABRE and ARE elements. These elements may be involved in the response to *S*. *sclerotiorum*. Among the 19 *BnaGRFs* which response to *S*. *sclerotiorum* inoculation, 14 (73.69%) were clustered into the non-ε group, this is almost half of the non-ε group members. This data implied that the members of non-ε group paly more important roles in *S*. *sclerotiorum* resistance response. Together, these results suggest that *BnaGRFs* might participate in the response to *S*. *sclerotiorum via* the JA and SA signaling pathways. It has been reported that SA and JA signaling pathways are involved in the plant resistance to *S*. *sclerotiorum* in *B*. *napus*, whereas the specific regulatory process remains unknown (Wu et al., 2016). A comprehensive study on the function of GRFs and their interacting proteins will help to elucidate the response processes, and these GRFs may be further applied as genetic resources for breeding S. sclerotiorum-resistant plants in *B*. *napus*.

## Conclusion

Overall, this study presents a comprehensive analysis of the *BnaGRF* gene family in rapeseed and provides evidence for their possible roles in the response to *S*. *sclerotiorum* infection. In total, 46 *BnaGRFs* were identified in the *B*. *napus* genome, and classified into the ε and non-ε groups. Analysis of *cis*-acting regulatory elements in *BnaGRF* promoters and evaluation of *BnaGRF* expression patterns in various tissues and under abiotic stresses revealed that these genes play important roles in plant development and stress responses. RNA-seq data showed that 41.30% of the *BnaGRFs* are involved in the response to *S*. *sclerotiorum* infection.

In summary, these findings provide a detailed characterization of *BnaGRFs* in *B*. *napus*, and could serve as a platform for the functional analysis and genetic improvement of agronomic traits in *B*. *napus*.

## Data availability statement

The datasets presented in this study can be found in online repositories. The names of the repository/repositories and accession number(s) can be found in the article/Supplementary material.

## Author contributions

QS and YPW designed the research. QS, YX, PL, YL, and YW performed the experiments. QS, YX, PL, and YL analyzed the data. QS, YX, and YPW wrote and revised the manuscript. All authors read and approved the current version of the manuscript.

## Footnotes

1 http://cbi.hzau.edu.cn/bnapus/index.php, accessed on 10 February 2021.

2
http://pfam.xfam.org/

3
https://smart.embl-heidelberg.de/

4
https://www.ncbi.nlm.nih.gov/Structure/cdd/cdd.shtml

5
https://www.genome.jp/tools/motif/

6
https://www.expasy.org/

7
https://wolfpsort.hgc.jp/

8
http://plants.ensembl.org/info/data/ftp/index.html

9
http://cbi.hzau.edu.cn/cgi-bin/rape/download_ext

10
http://www.genoscope.cns.fr/brassicanapus/data/

11
http://yanglab.hzau.edu.cn/BnTIR/eFP
