# Supplementary material for: Genome-wide analysis of the G-box regulating factors protein family reveals its roles in response to Sclerotinia sclerotiorum infection in rapeseed (Brassica napus L.)
Source: Front Plant Sci. 2022 Aug 12;13:986635. doi: 10.3389/fpls.2022.986635 (PMC9412199; doi:10.3389/fpls.2022.986635)
Supplement: Supplementary file 1 [file Presentation_1.PPTX]

## Slide 1
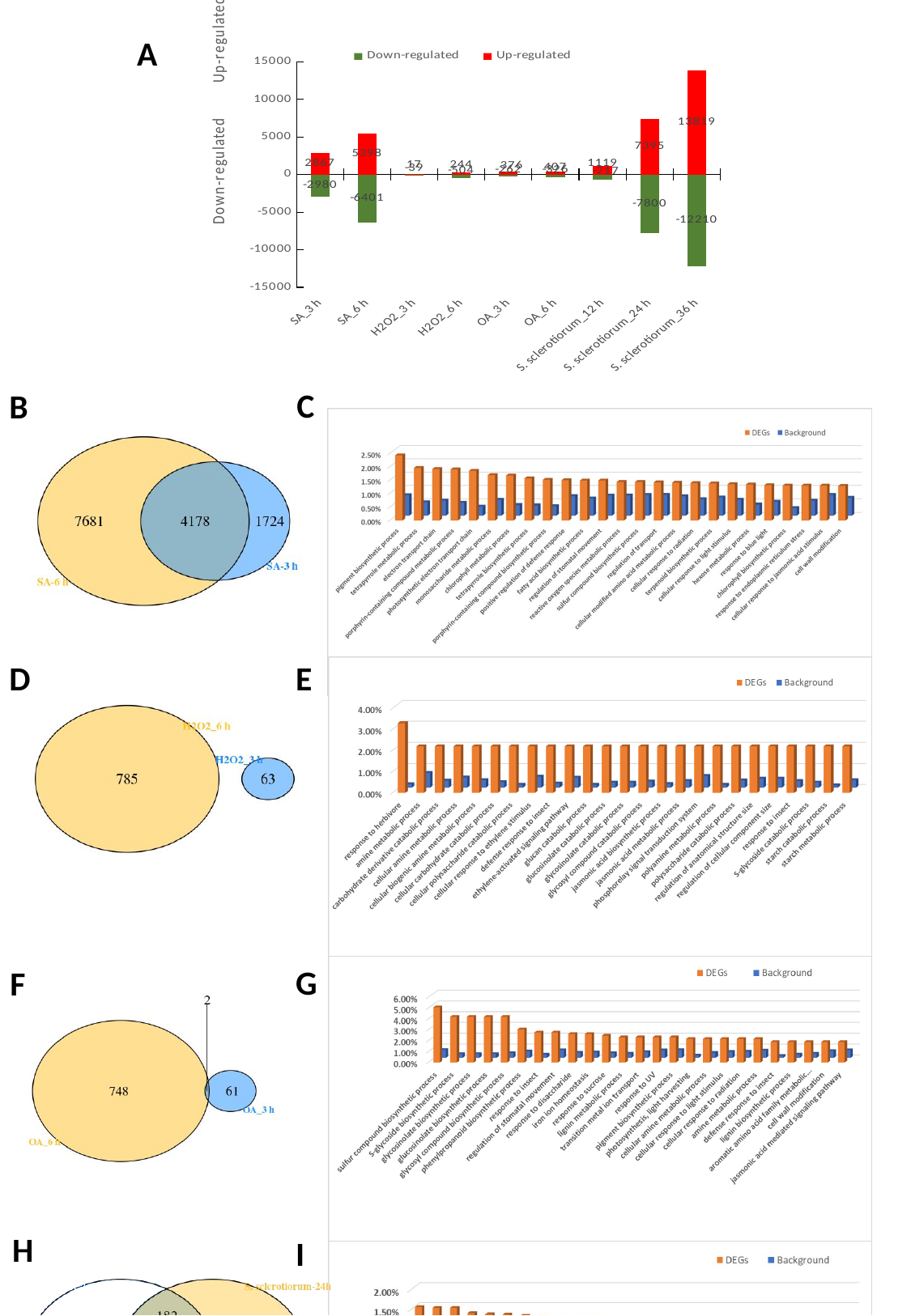

A
### Chart
| Category | Down-regulated | Up-regulated |
|---|---|---|
| SA_3 h | -2980.0 | 2867.0 |
| SA_6 h | -6401.0 | 5398.0 |
| H2O2_3 h | -39.0 | 17.0 |
| H2O2_6 h | -504.0 | 244.0 |
| OA_3 h | -262.0 | 376.0 |
| OA_6 h | -326.0 | 407.0 |
| S. sclerotiorum_12 h | -717.0 | 1119.0 |
| S. sclerotiorum_24 h | -7800.0 | 7395.0 |
| S. sclerotiorum_36 h | -12210.0 | 13819.0 |C
B
D
E
G
F
H
I
Figure S1 DEGs identification and GO enrichment analysis of DEGs that may be responsible for the SA, H2O2, OA, and Sclerotinia resistance. (A) The number of DEGs (differentially expressed genes) that were up- or down-regulated at different time points under different treatments. Venn diagram showing the DEGs expressed under the SA (B), H2O2 (D), OA (F), and S. sclerotiorum (H) treatment. The overlapping regions correspond to the number of DEGs present at more than one sampling point. (C, E, G, I) Biological process categorization of the RDEGs based on GO enrichment analysis. The y-axis is the percentage of genes mapped by the term, representing the abundance of the GO term. The percentage for the input list is calculated by the number of genes mapped to the GO term divided by the number of all genes in the input list. The same calculation was applied to the reference list (background) to generate its percentage. Terms in the top 25 are shown. The venn diagrams were drawn by online tools VennDetail Shiny App (http://hurlab.med.und.edu:3838/VennDetail/). The GO enrichment analysis were performed by BnIR:Brassica information resource (hzau.edu.cn).
